# Sphingolipid metabolism-related genes as diagnostic markers in pneumonia-induced sepsis: the AUG model

**DOI:** 10.1038/s41598-025-01150-8

**Published:** 2025-05-20

**Authors:** Jing Wu, Xiaomin Li, Zhihao Chen, Yiting Lin, Qiuyue Long, Mingzheng Jiang, Xiaoyi Hu, Shixu Song, Hongli Ye, Jiwei Li, Fangfang Wu, Jianshi Zheng, Minghui Wang, Zhancheng Gao, Pu Ning, Yali Zheng

**Affiliations:** 1 Department of Respiratory, Critical Care and Sleep Medicine, School of Medicine, Xiamen University, Xiang’an Hospital of Xiamen University, Xiamen, 361102 Fujian China; 2 Institute of Chest and Lung Diseases, Xiang’an Hospital of Xiamen University, Xiamen, 361102 Fujian China; 3 Department of Respiratory and Critical Care Medicine, Xiamen Haicang Hospital, Xiamen, 361026 Fujian China; 4 Department of Respiratory and Critical Care Medicine, Peking University People’s Hospital, Beijing, 100044 China; 5 Department of Pulmonary and Critical Care, The Second Affiliated Hospital of Xi’an Jiaotong University, Xi’an, 710000 Shanxi China; 6 Department of Cardiology, School of Medicine, Xiamen University, Xiang’an Hospital of Xiamen University, Xiamen, Fujian China

**Keywords:** Pneumonia-induced sepsis, Sphingolipid metabolism, Programmed cell death, MIF signaling, Computational biology and bioinformatics, Biomarkers, Infectious diseases, Respiratory tract diseases

## Abstract

Pneumonia-induced sepsis (PIS) is a life-threatening condition with high mortality rates, necessitating the identification of biomarkers and therapeutic targets. Sphingolipid, particularly ceramides, are pivotal in modulating immune responses and determining cell fate. In this study, we identified a novel gene signature related to sphingolipid metabolism, comprising *ACER3*, *UGCG*, and *GBA*, which are key enzymes involved in the synthesis and metabolism of ceramides. This signature, termed the “AUG model”, demonstrated strong diagnostic performance and modest prognostic efficacy across both training (GSE65682) and validation (E-MTAB-1548 and E-MTAB-5273) datasets. A clinical cohort comprising 20 PIS patients, 31 pneumonia cases, and 11 healthy controls further validated the increased expression of AUG genes at both mRNA and protein levels in peripheral blood samples upon admission. Our comprehensive analysis of bulk and single-cell transcriptome datasets revealed that these genes are implicated in immune cell death pathways, including autophagy and apoptosis. Additionally, cell-communication analysis indicated that enhanced macrophage migration inhibitory factor (MIF) signaling may be associated with dysregulated sphingolipid metabolism, potentially driving the inflammatory cascade. This study identifies a novel predictive model for PIS, highlighting the role of sphingolipid metabolism-related genes in disease progression and suggesting potential therapeutic targets for sepsis management.

## Introduction

Pneumonia remains a leading cause of global morbidity and mortality, imposing a significant healthcare burden with a mortality rate of 6.5% during hospitalization^1^. Sepsis, a life-threatening complication of pneumonia, is characterized by dysregulated systemic inflammatory response after infection, particularly in intensive care unit (ICU) patients^2^. The high mortality rate of patients with pneumonia-induced sepsis (PIS), which can reach up to 50%^3^, underscores the urgency for early diagnostic and prognostic markers. Early identification and stratification of sepsis is crucial for improving clinical outcomes and reducing mortality, thereby optimizing clinical management and mitigating the socioeconomic impact of this devastating condition.

Sphingolipids, integral to cellular membranes, are recognized as bioactive lipids involved in cell growth, differentiation, senescence, programmed cell death, and signaling transduction processes^4–7^. Recent studies have elucidated the connection between sphingolipid homeostasis and endothelial barrier function^8^. Specifically, dysregulation of sphingolipids, such as the accumulation of ceramides, has been shown to impair endothelial barrier integrity, leading to increased vascular permeability and subsequent acute lung injury (ALI)^9^. This endothelial dysfunction is a key event in the progression of PIS, where the systemic inflammatory response to infection leads to widespread organ dysfunction. Consistent with this, elevated serum levels of sphingosine-1-phosphate^9,10^, a sphingolipid metabolite, and ceramides^11^ have been observed in septic patients and are associated with disease prognosis. Our previous research also substantiates these findings; we demonstrated significantly dysregulated sphingolipid metabolism in pneumonia patients as early as admission, and some sphingolipids could serve as prognosis biomarkers^7,12^. Additionally, dysregulated sphingolipid metabolism has been intricately linked to the pathophysiology of various respiratory diseases, including bronchiectasis, chronic obstructive pulmonary disease, asthma, and cystic fibrosis^6,13,14^. These observations underscore the importance of sphingolipid metabolism in respiratory health, warranting further investigation into its potential as a disease marker or therapeutic target in PIS.

This study identified three key sphingolipid metabolism-related genes (*ACER3*, *UGCG*, and *GBA*), which could serve as diagnostic and prognostic indicators for PIS. The model, termed “the AUG model” for its components *ACER3*, *UGCG*, and *GBA*, exhibited high sensitivities in diagnosing and high specificities in 28-day mortality predicting in both validation datasets and clinical cohorts. Mechanistic exploration via public bulk and single-cell transcriptomic data suggested a potential role for the AUG genes in the programmed cell death of immune cells in PIS, especially apoptosis and autophagy. Further cell communication analysis revealed enhanced interactions between monocytes and other immune cells in the SEP-AUG^hi^ group, mediated through the MIF signaling pathway. These findings underscore the intricate link between deregulated sphingolipid metabolism and the MIF signaling in the hyperactivation of immune cells during sepsis. The study set the stage for developing targeted therapeutic interventions to modulate sphingolipid metabolism and MIF signaling, offering new strategies for PIS management.

## Methods

### Dataset download 

Transcriptomic microarray datasets of peripheral blood samples from patients with PIS were screened from the Gene Expression Omnibus (GEO) database^15,16^ and BioStudies database^17^. Finally, GSE65682 (as a training set), E-MTAB-1548, and E-MTAB-5273 (as validation sets) were obtained. GSE65682 contained 192 patients with PIS and 42 healthy controls. E-MTAB-1548 contained 82 patients with PIS and 15 healthy controls. E-MTAB-5273 contained 127 patients with PIS and 10 healthy controls. To ensure robust signature identification, the AUG model was constructed using a GEO microarray dataset (GSE65682) and independently validated using BioStudies RNA-seq datasets (E-MTAB-1548 and E-MTAB-5273). These two datasets were processed and analyzed separately, without technical integration or batch correction, to avoid artifacts and to better assess cross-platform reproducibility. A single-cell transcriptome analysis dataset (EGAD00001010927) of whole-blood leukocyte samples from 26 patients with sepsis and 6 healthy controls was acquired from the European Genome-Phenome Archive (EGA) (https://ega-archive.org/)^18^.

### Screening sphingolipid metabolism-related differentially expressed genes

We transformed the probe into a gene symbol in each dataset based on the platform’s annotation file; when multiple probes were mapped to the same gene symbol, the mean value of probes was selected as the gene expression value. Differentially expressed genes (DEGs) between PIS and control were analyzed via the “limma” package in R software, with the following cutoff criteria: a log2 fold change (log2 FC) >|0.5| and an adjusted *P* value (Adj.P.Val) < 0.05. Sphingolipid metabolism-related genes (n = 53) were extracted from the KEGG database^19^ (Supplementary Information 3. Table S1). The Venn plot visualized the intersection of DEGs and sphingolipid metabolism-related genes.

### Heatmaps and volcano plots

We used the “pheatmap” package to explore the differential high and low expression genes among the individuals with PIS and healthy controls. We used a volcano plot performed by the “ggplot2” package to exhibit significantly differentially expressed genes.

### Construction of a diagnostic model

Lasso-Cox regression was performed using the “glmnet” package of R software to identify which DEGs related to sphingolipid metabolism were associated with sepsis prognosis. The optimal lambda value was determined using tenfold cross-validation. We applied the LASSO-Cox model across a range of lambda values and selected λ = 4.8e−3, which yielded the highest mean concordance index (C-index) during tenfold cross-validation. This value corresponds to the optimal balance between model complexity and predictive performance. The final model identified three genes, ACER3, UGCG, and GBA, as the most robust prognostic signature. The C-index (concordance index) quantifies a model’s discriminative ability to correctly rank patient outcomes, with values ranging from 0.5 (no better than random chance) to 1.0 (perfect prediction). Higher values indicate better prognostic accuracy. Subsequently, we calculated the Diagnostic Score of each sample using this lambda value to obtain the final prognostic prediction model.

ROC Curve Analysis was used to evaluate the diagnostic accuracy of the model, which was expressed as the AUROC and 95% confidence interval (CI). The sensitivity and specificity for each set and clinical characteristic were calculated.

### Survival curve and the mortality rate

The individuals in the training set were divided into the AUG^hi^ and AUG^low^ groups by the median AUG value. The prognostic differences between the two groups were assessed using the “survival” package, with the log-rank test applied to determine the statistical significance of the survival rates. Subsequently, Kaplan–Meier (KM) survival curves were generated using the “survminer” package.

### GO and KEGG enrichment analysis

Gene ontology (GO) function analysis and Kyoto Encyclopedia of Genes and Genomes (KEGG) pathway enrichment analysis were performed using the “clusterProfiler” R package. The results were visualized by the ggplot2 package. *P* value < 0.05 was set as the cut-off criterion for significant enrichment.

### Single-cell transcriptome analysis

#### Unsupervised dimensional reduction and clustering

The gene expression matrices of each sample downloaded from the EGA database were imported into R (4.3.1) and transformed into Seurat objects using the “Seurat” package. Then the dataset was normalized using “LogNormalize” method in “Seurat” package with default parameters. In the next step, the “vst” method implemented in the “FindVariableFeatures” function was applied to find the top 4000 most variable genes. The data was subsequently subjected to principal component analysis (PCA), after which the uniform manifold approximation and projection (UMAP) algorithm was employed for visualizing the cell data, utilizing the top 50 principal components. Subsequently, graph-based clustering was conducted using the “FindClusters” function from the Seurat package on the PCA-reduced data. Cell identity annotation standards referred to the uploader’s instruction.

#### Gene set enrichment analysis (GSEA)

Gene set enrichment analysis was performed via the “clusterProfiler” R package. We calculated each gene set’s enrichment score (ES) and normalized enrichment score (NES), employing the FDR to determine significance levels. FDR < 0.25 and absolute normalized enrichment score (|NES|) > 1 were statistically significant. The results were visualized using the “dotplot” function within the “ClusterProfiler” package.

#### Cell–cell communication analysis

In the public single-cell transcriptome dataset, participants were stratified based on the median AUG scores. Sepsis patients were divided into SEP-AUG^hi^ and SEP-AUG^low^ groups, while healthy controls were grouped into HC-AUG^hi^ and HC-AUG^low^ accordingly. We imported single-cell RNA sequencing data into the CellChat package to explore the communication networks among various immune cells. Ligand-receptor networks for each cell type were constructed based on known ligand-receptor interactions in the database. These networks took into account not only the expression levels of the ligands and receptors but also the intricate interactions that occur between them. The CellChat pipeline was employed to calculate a communication score for each ligand-receptor pair, to assess the potential communications across different cell populations. In addition, sender and receiver scores for each cell population were determined, elucidating their roles in the overall communication network. The visualization tools provided by CellChat were used to display the cell communication network, including the connection weights and numbers between cell populations and the direction of communication. Salient ligand-receptor pairs were considered potential intercellular communication events.

### Human subjects enrollment and sample collection

The clinical cohort enrolled 20 patients with PIS, 31 patients with pneumonia as disease control, and 11 healthy controls at Xiamen University Xiang’an Hospital from July 3, 2022, to August 6, 2023. Supplemental Table S2 (Supplementary Information 3) provides detailed information about the 62 individuals. Participants were enrolled based on the following criteria: (1) Age ≥ 18 years; (2) Meeting Sepsis-3.0 consensus^20^; (3) Sepsis secondary to pneumonia; (4) Provision of written informed consent by participants or their legally authorized representatives; (5) Availability of complete baseline clinical data and verifiable follow-up records. The exclusion criteria included: (1) Coexisting chronic inflammatory or autoimmune diseases; (2) History of immunosuppressive therapy; (3) Death or discharge within 24 h of ICU admission. The peripheral blood samples were collected within 72 h of admission. The whole blood leukocytes were EDTA-anticoagulated and stored at − 80 °C until further analysis. The serum samples were separated and stored at − 80 °C. The Ethics Institute of Xiamen University Xiang’an Hospital (Approval No. XDYX202302K06) approved the study, and the study itself was conducted in accordance with the Declaration of Helsinki. All participants or their guardians provided written informed consent before enrollment.

### RNA extraction and quantitative real-time polymerase chain reaction (RT-qPCR)

Total RNA was isolated from the whole blood samples using TRIzol reagent (Invitrogen), and cDNA was synthesized from 500ng of total RNA using the Evo M-MLV RT Kit with gDNA Clean for qPCR (Accurate Biology). Each cDNA sample was diluted by a factor of 10, and a 5.5 μl aliquot was employed in a 12 μl PCR reaction mixture, which consisted of the SYBR Green Premix Taq HS qPCR Kit (Accurate Biology) and gene-specific primers. All the reactions were run in triplicates. Relative mRNA expression levels were normalized against the endogenous housekeeping gene, β-actin, and quantified using the 2 − ΔΔCT method. Primers for *UGCG*, *ACER3*, *GBA* and β-actin were obtained from Tsingke Biotechnology. The sequences of primers used for gene amplification were listed in supplemental Table S3 (Supplementary Information 3).

### Enzyme-linked immunosorbent assay (ELISA)

ELISA was employed to detect serum levels of *ACER3*, *UGCG*, and *GBA* across three groups: HC, pneumonia, and PIS groups. ELISA kits from ABclonal Technology were employed following the manufacturer’s protocol, utilizing a standard curve for accurate measurement. Briefly, 100 μl of serum samples from each group were added to respective wells of a 96-well plate. After further incubation with biotin-conjugated antibody and streptavidin-HRP, color development was achieved with TMB substrate, and the reaction was stopped for absorbance measurement at 450 nm using a microplate reader (Thermo Fisher Scientific).

### Statistical analysis

Statistical analysis and visualization of the datasets were performed using R software (version 4.3.1). Comparisons among clinical sample groups were performed using GraphPad Prism software (version 8.0.2). Normally distributed data were analyzed using one-way ANOVA followed by Tukey’s multiple comparisons test. For non-normally distributed data, the Kruskal–Wallis test was used, followed by pairwise comparisons with the Bonferroni-corrected Mann–Whitney test. All *p*-values were two-sided, with *p* < 0.05 indicating statistical significance. Error bars in figures represent 95% confidence intervals or one standard deviation as indicated.

## Results

### Development of a diagnostic model for PIS utilizing sphingolipid metabolism-related DEGs

**Fig. 1:**
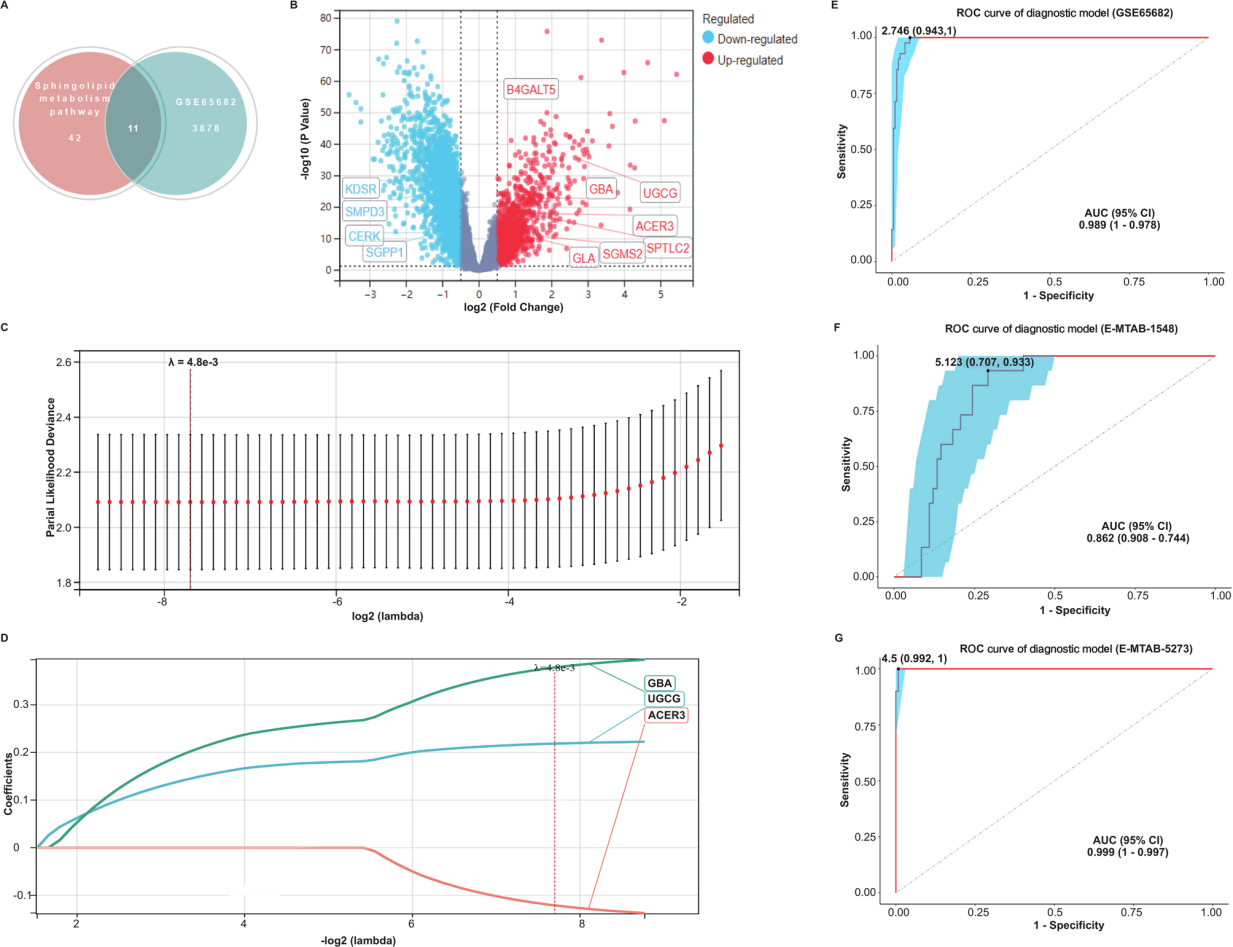
Construction and validation of a diagnostic model for pneumonia-induced sepsis. (**A**) The Venn diagram illustrates the overlap between the 53 sphingolipid metabolism-related genes from the KEGG database and the 3889 DEGs identified from the GSE65682 dataset. The threshold for identifying DEGs was set based on an adjusted P value (Adj.P.Val) < 0.05 and a |log2 FC|> 0.5. (**B**) Volcano plot of DEGs in the GSE65682 dataset. The eleven sphingolipid metabolism-associated DEGs were pointed out, with seven upregulated and four downregulated in the pneumonia-induced sepsis (PIS) patients. (**C**, **D**) Screening of variables based on Lasso regression. (**C**) The variation characteristics of the coefficient of variables. (**D**) The optimal value of the parameter lambda in the Lasso regression model, 4.8e−3, was selected using the cross-validation method. With lambda set to 4.8e−3, three genes—*ACER3*, *UGCG*, and *GBA*—were identified, which were then used to construct the diagnostic model, named the “AUG”model. (**E**) Validation of the diagnostic model in the GSE65682 dataset. (**F**) Validation of the diagnostic model in an external dataset E-MTAB-1548. (**G**) Validation of the diagnostic model in an external dataset E-MTAB-5273.

We performed a differential expression analysis on the GSE65682 dataset and identified 3889 DEGs, with 1289 up-regulated and 2600 down-regulated in PIS patients. The intersection analysis revealed 11 sphingolipid metabolism-related DEGs (Fig. 1A). Among these, seven genes, *SGMS2*, *ACER3*, *B4GALT5*, *SPTLC2*, *UGCG*, *GLA*, and *GBA*, were highly expressed in PIS cases, while the other four genes, *SGPP1*, *CERK*, *KDSR*, and *SMPD3*, were lowly expressed (Fig. 1B).

To construct a diagnostic model, we applied LASSO regression analysis to refine the selection of genes for a diagnostic model. The determination of optimal lambda (λ) and regression coefficient of each gene were illustrated in Fig. 1C. The probability of deviance was minimized when λ reached a minimum value. As shown in Fig. 1D, the best performance of the model was reached when three genes, *ACER3*, *UGCG*, and *GBA*, were included. The diagnostic score was calculated using the following formula: Diagnostic Score = 0.22 × exp*UGCG* + 0.38 × exp*GBA − *0.12 × exp*ACER3*.

The diagnostic accuracy of the model, termed “the AUG model” for its components *ACER3*, *UGCG*, and *GBA*, was evaluated using the area under the receiver operating characteristic curve (AUROC). In the GSE65682 dataset, the AUROC was 0.989 (95% CI 1–0.978) with a sensitivity and specificity of 1 and 0.943, respectively (Fig. 1E). The efficacy of the AUG model was further validated in two external datasets. In the E-MTAB-1548 dataset of PIS and HC, the model exhibited an AUROC of 0.826 (95% CI 0.908–0.744), with sensitivity and specificity of 0.933 and 0.707, respectively (Fig. 1F). In the E-MTAB-5273 dataset, comprising PIS individuals and HC, the AUROC was 0.999 (95% CI 1–0.997), with a sensitivity of 1 and a specificity of 0.992 (Fig. 1G). The finding indicated that the AUG model could serve as a diagnostic model for PIS.

### Prognostic abilities of the AUG model for 28-day mortality in PIS

**Fig. 2:**
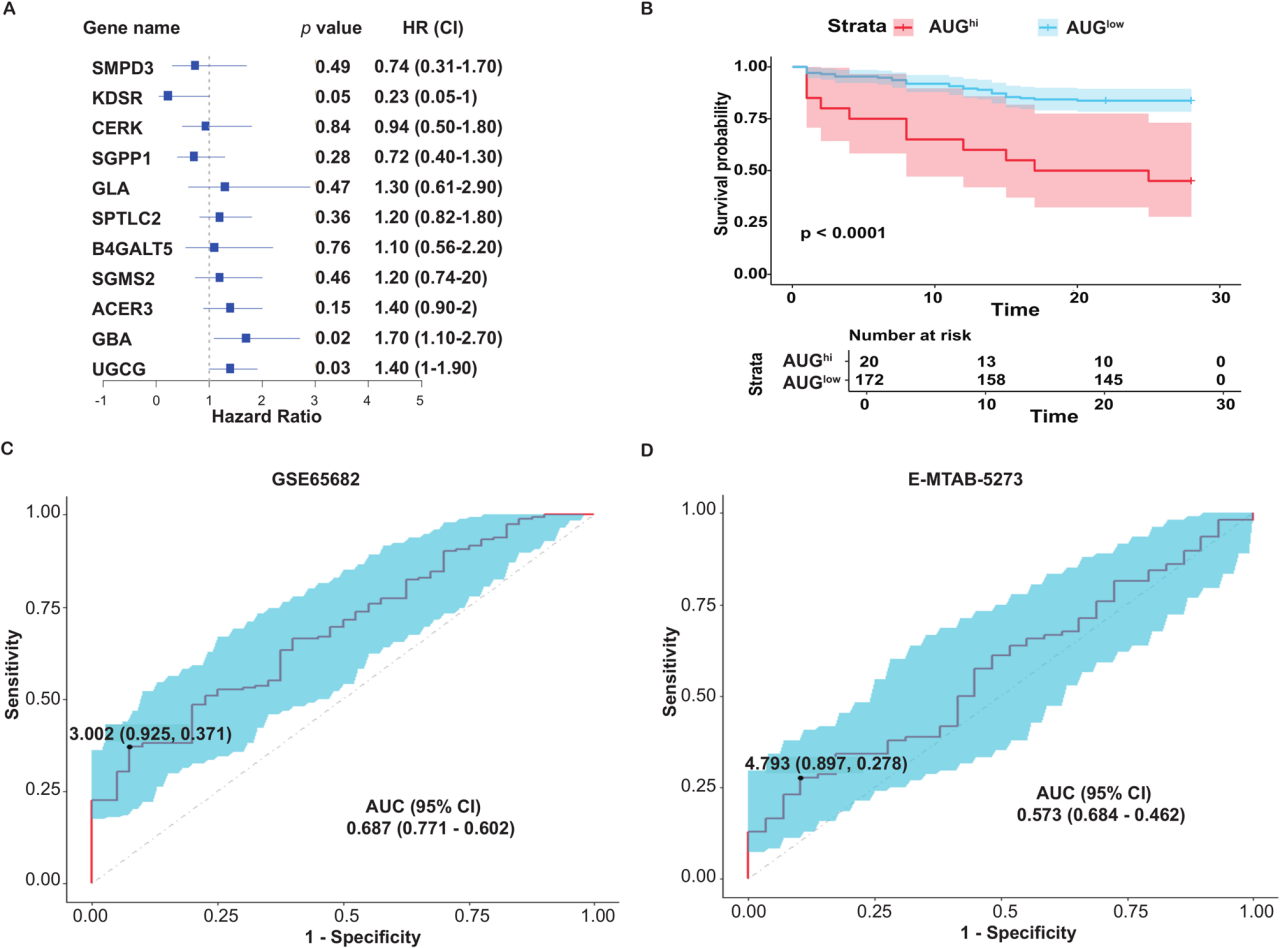
Prognostic performance of the AUG model in PIS patients. (**A**) The hazard ratio for the 11 sphingolipid-related DEGs associated with 28-day mortality in PIS patients. (**B**) Kaplan–Meier survival curves of 28-day mortality in the training set, stratified by the median “AUG” value. (**C**) ROC curve for 28-day survival status in the training set. The AUROC is 0.687 (95% CI 0.771–0.602), with a sensitivity of 0.371 and a specificity of 0.925. (**D**) ROC curve for 28-day survival status in the E-MTAB-5273 dataset. The AUROC is 0.573 (95% CI 0.684–0.462), with a sensitivity of 0.278 and a specificity of 0.897.

Multiple regression analysis revealed that within the eleven sphingolipid metabolism-associated genes, *UGCG* (Hazard Ratio with 95% CI, 1.4, 1–1.9, *P* = 0.03), *GBA* (HR 1.7, 1.1–2.7, *P* = 0.016) and *KDSR* (HR 0.23, 0.053–1, *P* = 0.053) were significantly associated with 28-day mortality in PIS patients (Fig. 2A). The prognostic relevance of the AUG model for predicting 28-day mortality in PIS was examined using the GSE65682 training set. Patients were categorized into AUG^hi^ and AUG^low^ groups based on the median score of the AUG model. Kaplan–Meier (KM) curves revealed significantly reduced survival in the AUG^hi^ group (*P* < 0.0001, Fig. 2B). As shown in Fig. 2C, ROC analysis yielded an AUROC of 0.687 (95% CI 0.771–0.602), with a sensitivity of 0.371 and a specificity of 0.925, suggesting the model’s predictive accuracy. Validation on the E-MTAB-5273 dataset produced an AUROC of 0.573 (95% CI 0.684–0.462), with specificity and sensitivity of 0.278 and 0.897, respectively (Fig. 2D). KM curve analysis was not performed due to the absence of detailed survival time data in E-MTAB-5273. Likewise, detailed survival information was missing in the E-MTAB-1548 dataset.

### Clinical validation of the AUG model

The efficacy of the AUG model was further validated in a clinical cohort. The cohort consisted of 62 individuals, including PIS patients (n = 20), pneumonia patients (n = 31), and healthy controls (n = 11). Demographic characteristics of the enrolled cases were detailed in supplemental Table S2 (Supplementary Information 3). Briefly, there were 12 (60%) males and 15 (75%) individuals over 60 years old in the PIS group. No significant differences were observed among the three groups.

**Fig. 3:**
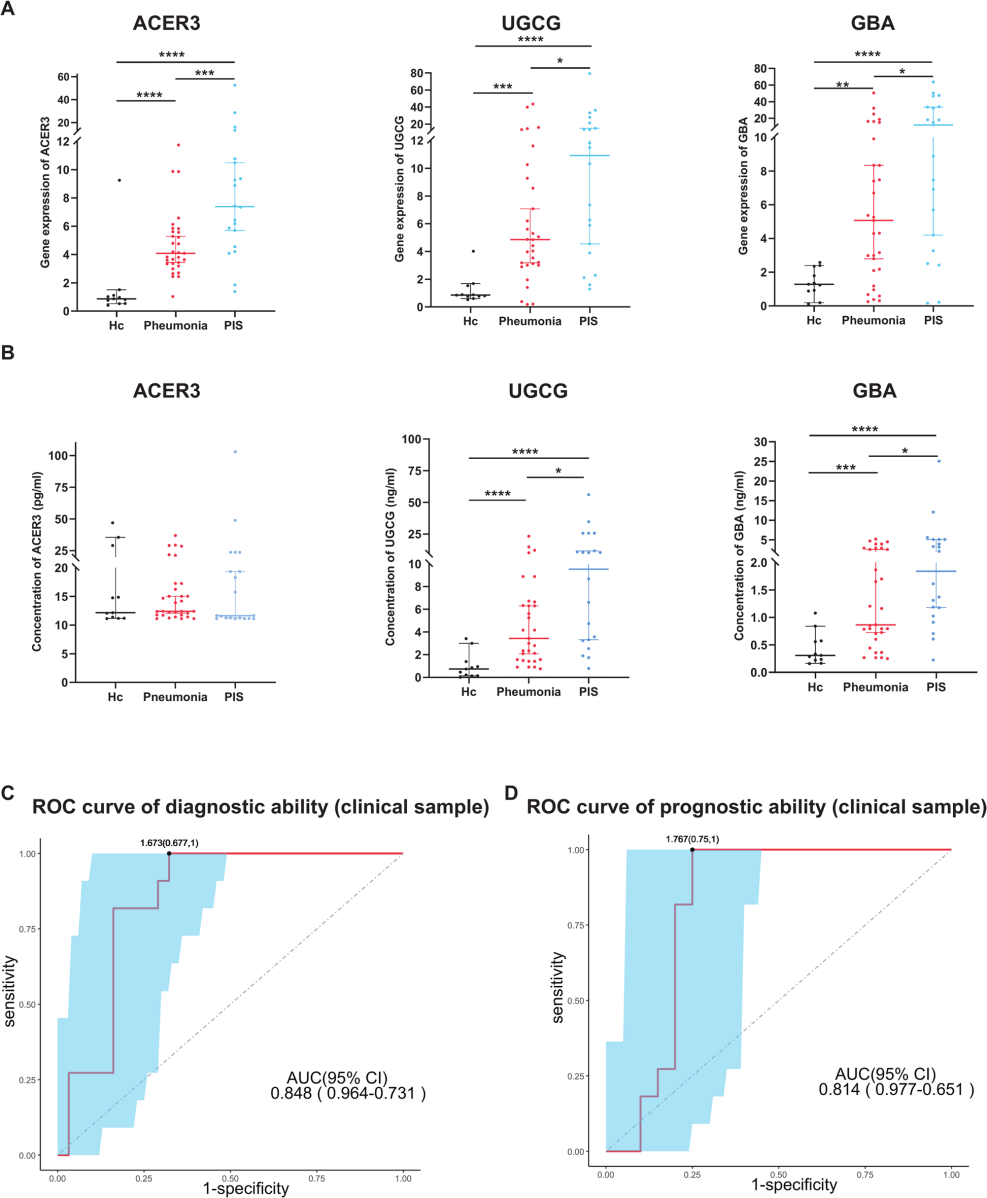
AUG Model Efficacy in PIS Cohort. (**A**) The mRNA expression levels of *ACER3*, *UGCG*, and *GBA* in peripheral blood leukocytes among three groups, including pneumonia patients (n = 31), PIS patients (n = 20), and healthy controls (HC, n = 11). Error bars throughout all figures represent a 95% confidence interval or one standard deviation where indicated. Data were analyzed using Bonferroni-adjusted Mann–Whitney tests for all intergroup comparisons (HC vs Pneumonia vs PIS), with significance denoted as *p < 0.05, **p < 0.01, ***p < 0.001, ****p < 0.0001. (**B**) Serum protein levels of *ACER3*, *UGCG*, and *GBA* in the three groups. Error bars throughout all figures represent a 95% confidence interval or one standard deviation where indicated. Data were analyzed using Bonferroni-adjusted Mann–Whitney tests for all intergroup comparisons (HC vs Pneumonia vs PIS), with significance denoted as *p < 0.05, **p < 0.01, ***p < 0.001, ****p < 0.0001. (**C**) Diagnostic performance of the AUG model (the diagnostic model based on three key genes: *ACER3*, *UGCG*, and *GBA*) in the clinical cohort, with an AUROC of 0.848 (95% CI 0.964–0.731), a sensitivity of 1, and a specificity of 0.677. (**D**) Prognostic performance of the AUG model for 28-day mortality in the clinical cohort, with an AUROC of 0.814 (95% CI 0.977–0.651), a sensitivity of 1, and a specificity of 0.75.

We quantified the mRNA expression levels of AUG in human peripheral blood leukocyte samples via RT-qPCR. As depicted in Fig. 3A and supplemental Table S4 (Supplementary Information 3), *ACER3*, *UGCG*, and *GBA* were significantly elevated in both pneumonia and PIS groups relative to the healthy controls (*P* < 0.05). Furthermore, the PIS group demonstrated a significantly higher upregulation in these gene expressions compared to the pneumonia cohort. Aligning with the RT-qPCR results, *UGCG* and *GBA* serum levels were significantly higher in the pneumonia and PIS groups compared to the healthy controls, with the PIS group showing a pronounced increase that reached statistical significance over the pneumonia group (*P* < 0.05) No significant differences were observed in the serum levels of *ACER3* among the three groups (Fig. 3B and supplemental information 3. Table S5).

Further analysis was conducted to evaluate the AUG model’s diagnostic and prognostic performance. ROC analysis yielded an AUROC of 0.848 for PIS (95% CI 0.964–0.731), with a sensitivity of 1 and a specificity of 0.677 (Fig. 3C). Furthermore, the AUROC for predicting the 28-day mortality was 0.814 (95% CI 0.977–0.651), with a sensitivity of 1 and a specificity of 0.75 (Fig. 3D). The data indicated that the AUG model effectively diagnoses and predicts outcomes for PIS patients.

### Functional analysis revealed that AUG genes were involved in the procession of cell death

**Fig. 4:**
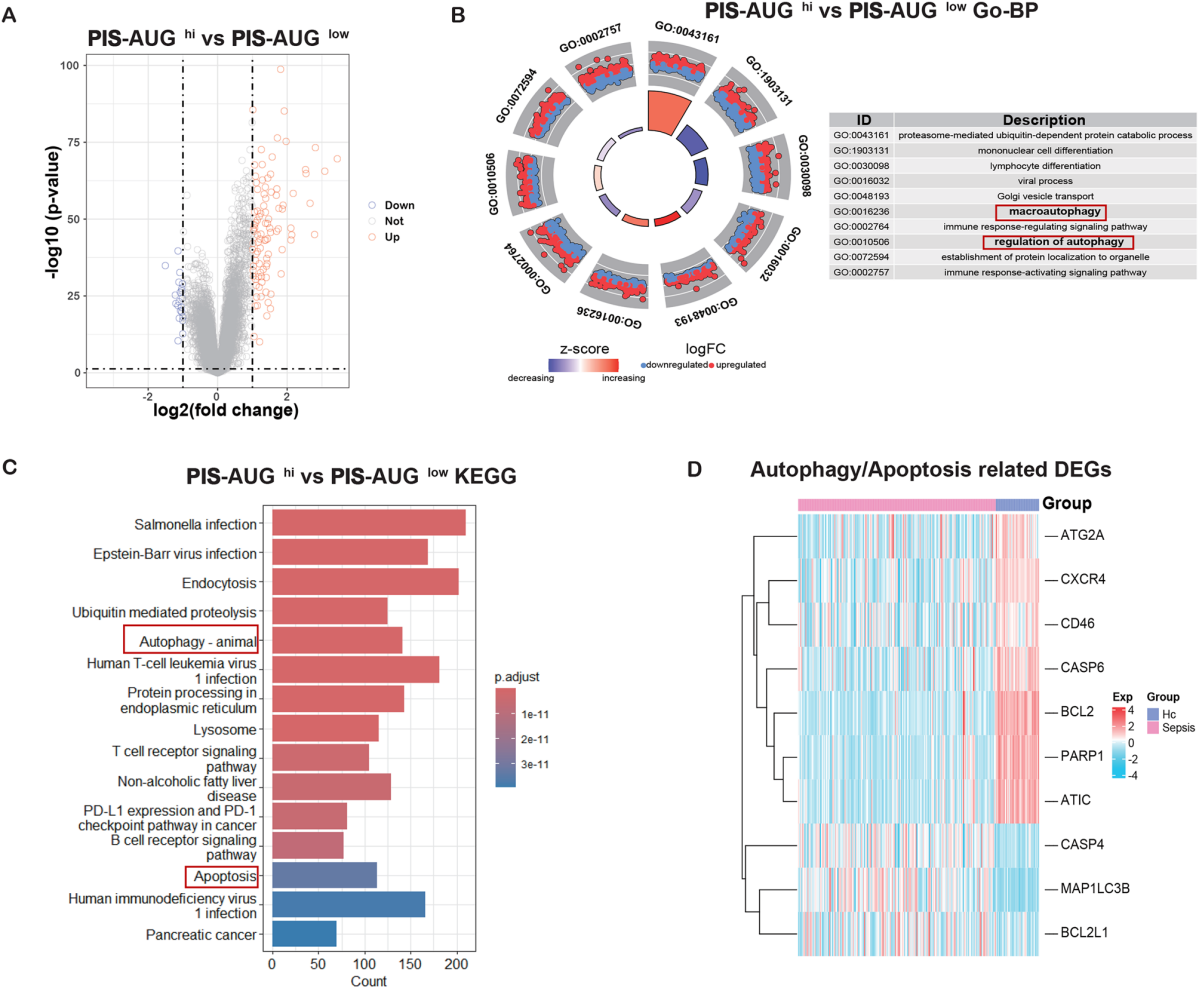
Bulk transcriptomic analysis revealed that AUG genes were involved in cell death signaling pathways. (**A**) Volcano plot revealing DEGs in PIS-AUG^hi^ versus PIS-AUG^low^ patients. The threshold was set at |logFC|> 1, *P* < 0.05. (**B**) Gene Ontology (GO) analyses of DEGs. (**C**) KEGG enrichment analysis of DEGs. Pathways related to cell death, including apoptosis and autophagy, were highlighted with red boxes in Figures B and C. (**D**) Heatmap demonstrating mRNA expression levels of genes associated with autophagy and apoptosis in PIS (pink) and healthy controls (HC) (blue).

To delve deeper into the mechanisms by which *UGCG*, *GBA*, and *ACER3* influence the pathology of PIS, we conducted a functional enrichment analysis. First, PIS patients in the GSE65682 dataset were stratified into two groups based on the median score of the AUG model: a high-expression group (AUG^hi^) and a low-expression group (AUG^low^). A total of 117 DEGs were identified between the two groups (Fig. 4A). Subsequent GO analysis highlighted that the AUG^hi^ group was significantly enriched in pathways related to immune cell differentiation and autophagy (Fig. 4B). In addition, KEGG enrichment analysis revealed that the cell death signaling pathways, including autophagy and apoptosis, were enriched in the AUG^hi^ group (Fig. 4C). In parallel, we generated a heatmap to visually represent the expression patterns of genes associated with autophagy and apoptosis within the PIS and healthy control groups (Fig. 4D). These findings suggested that autophagy, a critical biological process, may play a central role in the progression of PIS.

### Cellular expression profiling of AUG genes in sepsis patients

**Fig. 5:**
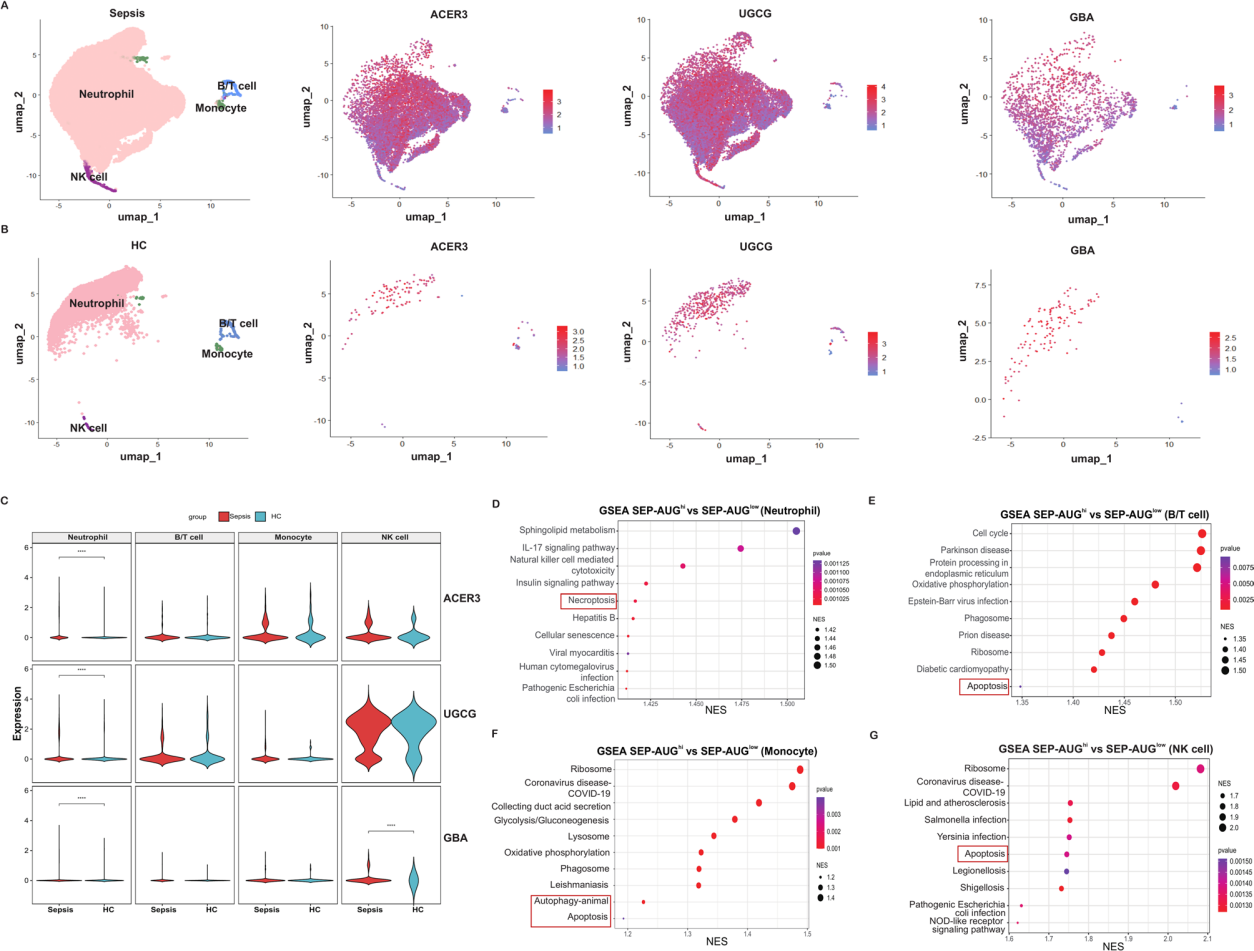
Single-cell expression profiling of AUG genes in patients with sepsis. (**A**, **B**) Single-cell transcriptome analysis of AUG gene distribution among immune cell types in sepsis patients (**A**) and healthy controls (HC) (**B**). Cell types were indicated by color: pink for neutrophils, blue for B/T cells, green for monocytes, and purple for NK cells. (**C**) Comparative quantitative analysis of AUG gene expression levels in different immune cell types between sepsis patients and HC groups. (**D**–**G**) Gene Set Enrichment Analysis (GSEA) of DEGs between the SEP-AUG^hi^ and SEP-AUG^low^ groups revealed enriched pathways associated with the AUG genes in neutrophils (**D**), B/T cells (**E**), monocytes (**F**), and NK cells (**G**), respectively.

Given the current absence of single-cell datasets specifically for PIS patients, we subsequently analyzed the cellular expression patterns of AUG genes using a sepsis patient single-cell dataset (EGAD00001010927) as the closest available proxy. After quality control and removal of doublets, four major immune cell subgroups were clustered and annotated based on the canonical annotation of marker genes for immune cells, including neutrophil cells, B/T cells, monocytes, and NK cells. Neutrophil cells were identified by the expression of *FCGR3A* and *FCGR3B*, B/T cells by *CD45*, monocytes by *CD14* and *CD163*, NK cells by *GNLY* and *KLRD1* (Fig. 5A, B). The expression patterns and levels of the AUG genes across these cell types were examined. As shown in Fig. 5A, B, the AUG gene expression was pervasive across all cell subpopulations in both sepsis patients and healthy controls (HC). Quantitative analysis of gene expression across two groups revealed that the expression of the AUG genes in neutrophils was significantly diminished in the HC group, as was the expression of *GBA* in NK cells (Fig. 5C). Next, sepsis patients were classified into high (SEP-AUG^hi^) and low (SEP-AUG^low^) expression groups based on the median of the AUG model score. Gene Set Enrichment Analysis (GSEA) were conducted to explore the AUG function in different cell types (Fig. 5D–G). Consisting with bulk analysis (Fig. 4B, C), the result also suggested that the role of the AUG genes in sepsis is associated with programmed cell death, including necroptosis, autophagy, and apoptosis. To elaborate, neutrophils in the SEP-AUG^hi^ group were associated with necroptosis (Fig. 5D), and monocytes were involved in the autophagy and apoptosis pathways (Fig. 5F). In contrast, B/T cells (Fig. 5E) and NK cells (Fig. 5G) demonstrated enhanced apoptosis activities. Collectively, the expression patterns of AUG genes revealed a distinct alteration in gene expression that is correlated with sepsis. This alteration was likely associated with immune cell deaths, playing a significant role in the pathophysiological progression of the disease.

### Enhanced MIF signaling pathway in AUG^hi^ sepsis (SEP-AUG^hi^) patients

**Fig. 6:**
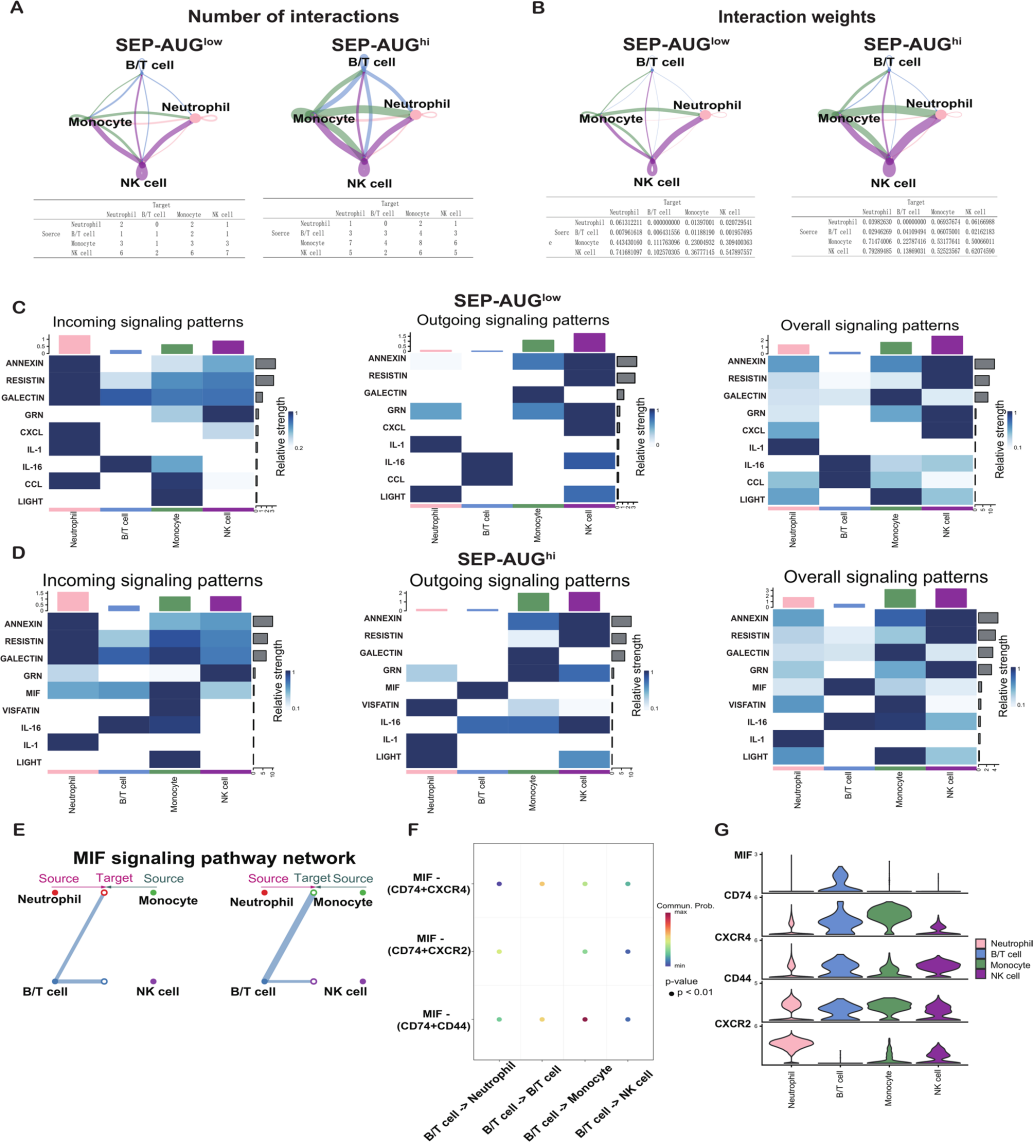
MIF signaling mediated enhanced immune cell communications in sepsis patients with high AUG expression. (**A**, **B**) Analysis of intercellular communication numbers (**A**) and weights (**B**) in PIS patients with low (SEP-AUG^low^) and high (SEP-AUG^hi^) AUG expression levels. Arrows denoted the direction of signal transmission from signaling to receiving cells. (**C**, **D**) Comparative analysis of incoming, outgoing, and overall signaling patterns across different cell subsets in SEP-AUG^low^ (**C**) and SEP-AUG^hi^ groups (**D**). Upper square bar graphs indicated communication strength within specific pathways, while grey bar graphs represented the number of receptor-ligand pairs. (**E**) Communication signals within the MIF signaling pathway among various immune cell types, with line thickness reflecting signal strength. (**F**) Contribution of individual ligand-receptor pairs to the MIF signaling pathway across different cell types, color-coded by P-value significance. (**G**) Expression distribution of MIF signaling pathway markers, including *MIF*, *CD74*, *CXCR4*, *CD44*, and *CXCR2*, in distinct cell subpopulations.

To elucidate the cellular crosstalk in sepsis, we employed CellChat for a comprehensive cell–cell interaction analysis. Compared to the SEP-AUG^low^ group, the interaction weights and numbers between immune cells were all significantly increased in the SEP-AUG^hi^ group during sepsis, especially between monocytes and other immune cells (Figs. 6A, B). while in healthy status, the most strong cellular communication was observed between NK cells and other immune cells in the HC-AUG^hi^ group, compared to the HC-AUG^low^ group (supplemental Figure S1A, B). These findings underscore the augmented role of AUG in modulating the signaling of monocytes and other immune cells during sepsis. Further analysis revealed that the SEP-AUG^hi^ group exhibited specifically enhanced macrophage migration inhibitory factor (MIF) signaling, compared to the SEP-AUG^low^ group (Fig. 6C). In contrast, upregulated MIF signaling was not observed in both HC-AUG^hi^ group or HC-AUG^low^ (Fig. 6C and D) groups. These findings suggest that the activation of the MIF signaling pathway in the SEP-AUG^hi^ group is specifically associated with sepsis pathogenesis, rather than being a general feature of high AUG expression. Analysis of MIF gene expression in our clinical cohort revealed significantly elevated levels in PIS patients compared to healthy controls (HC) (Figure S1E), providing further evidence for a direct association between MIF signaling and disease severity.

The hierarchical plot demonstrated that the MIF signal predominantly emanates from B/T cells and is received by monocytes (Fig. 6E). The ligand-receptor pairs within different cell types were analyzed, and *CD74/CD44* was identified as the key ligand-receptor pair facilitating the MIF signaling between B/T cells and monocytes (Fig. 6F). Furthermore, we quantified the expression levels of signature genes involved in the MIF signaling pathway (including *MIF*, *CD74*, *CXCR4*, *CD44*, and *CXCR2*) across the immune cells. As shown in Fig. 6G, *MIF* was predominantly expressed by B/T cells, while *CD74* and *CD44* were majorly expressed in B/T cells and monocytes. These results highlighted the intensified communication between monocytes and other immune cells through MIF signaling in the SEP-AUG^hi^ group, suggesting that MIF signaling may be a critical pathway driving immune cell hyperactivation in sepsis.

## Discussion

In the current study, we identified three critical enzymes (*ACER3*, *UGCG*, and *GBA*) and developed a diagnostic and prognostic predictive model for pneumonia-induced sepsis (PIS), termed the “AUG model”. The AUG model demonstrated strong diagnostic performance and modest prognostic efficacy across training, validation, and real-world clinical datasets, reinforcing its clinical applicability. Functional analysis using public bulk and single-cell transcriptome data indicated that the AUG genes may be involved in programmed cell death of immune cells, contributing to the pathophysiological progression of PIS. Additionally, cell communication analysis revealed intensified monocyte signaling via the MIF pathway in the SEP-AUG^hi^ group. In summary, the study presents a novel and effective diagnostic and prognostic model for PIS, underscoring the potential relationship between dysregulated sphingolipid metabolism and dysregulated programmed immune cell deaths, revealing the crucial role of MIF signaling in immune cell hyperactivation during sepsis, which paves the way for targeted therapeutic strategies.

Our findings is in line with the established role of sphingolipid metabolism in modulating cell death mechanisms across diverse cell types, including neuronal cell parthanatos^21^, cardiomyocyte apoptosis^22^, and apoptosis and necroptosis in cancer cells^23^. The individual roles of the AUG genes in cell death are also well-documented. *ACER3*, alkaline ceramidase 3, induces apoptosis in various endothelial and cancer cells^24^. *UGCG*, UDP-glucose ceramide glucosyltransferase, inhibition significantly enhances cell death in tumor cells^25,26^. *GBA*, glucocerebrosidase, is a key mediator of autophagic cell death and contributes to neuronal degeneration via autophagy-lysosomal dysfunction^27,28^. In PIS, the upregulated expression of AUG genes is associated with poor prognosis, suggesting that dysregulated sphingolipid metabolism may contribute to immune cell apoptosis or autophagy, potentially exacerbating immune suppression, tissue damage, and organ failure. Prior research has established the prognostic significance of apoptosis-related factors in sepsis, particularly emphasizing their role in tissue damage and organ failure^29,30^. Excessive apoptosis of immune cells has been shown to contribute to severe immune suppression in the late stages of sepsis^31^. Furthermore, enhanced autophagy in sepsis can activate aberrant macrophages, thereby modulating immune responses^32^. Consequently, we postulate that the AUG genes may significantly influence disease progression through the modulation of autophagy and apoptosis in immune cells.

Ceramide, as a central molecule in sphingolipid metabolism, has been proven in numerous studies to induce a state of high inflammation in the human body^33–35^ and promote cell death^26^, leading to multi-organ dysfunction. Elevated levels of ceramide are also considered a risk factor in sepsis patients^11^. In the sphingolipid metabolic process, *ACER3* is responsible for hydrolyzing ceramide into dihydroceramide, while *UGCG* participates in the first step of synthesizing sphingoglycolipids from ceramide^36,37^. These two enzymes might serve to synthesize glycosphingolipids and sphinganine from ceramide, thereby exacerbating the hyperinflammatory state observed in PIS. In contrast to *UGCG*, *GBA* catalyzes the hydrolysis of glucosylceramide into free ceramide and glucose, while also being involved in the synthetic pathway of ceramide^36,37^. We hypothesize that in sepsis, GBA primarily functions by promoting the synthesis of sphingosine, which serves as a precursor for the synthesis of ceramide through the salvage pathway. Our explanation of the AUG model’s mechanism of action further emphasizes the importance of ceramide in the pathophysiological process of sepsis, offering a potential avenue for therapeutic intervention by targeting the sphingolipid metabolic pathway. However, the specific mechanisms underlying these processes require further extensive experimental validation and exploration.

We delineate the significant involvement of MIF signaling in immune cell communications within the AUG^hi^ group, suggesting its role as a key mediator of immune cell hyperactivation in PIS. MIF, a pleiotropic cytokine secreted by various cellular sources, is increasingly recognized for its central role in the pathogenesis of inflammatory conditions and oncogenesis. Previous studies have identified MIF as a potential biomarker and therapeutic target for acute infection^38^ and sepsis^39^, with its expression levels correlating with sepsis incidence and mortality rates^40^, as well as in Streptococcus pneumoniae infections^41^. The therapeutic potential of MIF inhibition is further supported by studies demonstrating its efficacy in mitigating inflammatory diseases and sepsis-induced acute lung injury in preclinical models^38,40,42,43^. Moreover, a link between MIF and sphingolipid metabolism has been previously established. Sphingolipids can impact tumor prognosis and neurodegenerative disorder progression by modulating the nuclear translocation of MIF molecules or MIF expression in tumor cells^23^, neurons^21^, and myeloid cells^44^. However, the intersection between the sphingolipid metabolism pathway and MIF signaling in the context of sepsis has not been extensively explored. The enhanced communications between lymphocytes and monocytes observed in our study via the CD74/CD44 ligand-receptor pair suggest a potential cascade reaction that drives immune cell hyperactivation in PIS. These findings provide novel insights into the intricate interplay between MIF signaling and sphingolipid metabolism in PIS, offering a foundation for future studies to unravel the multifaceted mechanisms underlying immune cell death and identify potential therapeutic targets.

There are several limitations inherent in our research. First, while our AUG model demonstrated significant predictive capabilities, it was primarily derived from bioinformatics analysis of existing datasets. Moreover, the clinical cohort used for validation in our study was relatively small and may not fully represent the broader patient population. large-scale, multicenter clinical trials are necessary to enhance the generalizability of our results. Secondly, although we identified an association between the upregulation of the AUG genes and immune cell apoptosis or autophagy, our study did not establish a direct causal link. The functional implications of these genes in sepsis were inferred from existing literature and our bioinformatics analysis. This suggests the need for experimental validation, potentially through in vitro or in vivo models. Lastly, our study focused on three key enzymes within the sphingolipid metabolism pathway. While our study suggests that targeting sphingolipid metabolism could be a potential therapeutic strategy, the translational potential of this approach was not directly explored. Future studies should investigate the therapeutic modulation of *ACER3*, *UGCG*, and *GBA* to assess their direct impact on sepsis outcomes. In parallel, integrative analyses incorporating other metabolic pathways will be necessary to comprehensively understand the broader metabolic reprogramming in PIS. Despite these limitations, our work provides a foundation for future investigations into the role of sphingolipid metabolism in sepsis and the potential for targeted therapies.

## Conclusion

Our findings advance the understanding of the molecular signatures of PIS but also pave the way for the development of targeted therapies aimed at modulating the sphingolipid metabolic pathway and MIF signaling, thus offering new avenues for improving clinical management and patient outcomes in sepsis.

## Supplementary Information

Supplementary Information 1.

Supplementary Information 2.

Supplementary Information 3.

## Data Availability

This article contains supplemental data. The data supporting this study’s findings are available within the article and its supplementary materials. Raw data are available from the corresponding authors upon reasonable request.

## Abbreviations

PIS: Pneumonia-induced sepsis
MIF: Macrophage migration inhibitory factor
ACER3: Alkaline ceramidase 3
GBA: Glucocerebrosidase
UGCG: UDP-glucose ceramide glucosyltransferase
DEGs: Differentially expressed genes
GEO: Gene Expression Omnibus
CI: Confidence interval
GO: Gene ontology
KEGG: Kyoto encyclopedia of genes and genomes
GSEA: Gene set enrichment analysis
RT-qPCR: Quantitative real-time polymerase chain reaction
ELISA: Enzyme-linked immunosorbent assay
PBS: Phosphate-buffered saline
AUROC: The area under the receiver operating characteristic curve
HC: Healthy controls
SEP: Pneumonia-induced sepsis patients
