# Supplementary figures and images for: Sphingolipid metabolism-related genes as diagnostic markers in pneumonia-induced sepsis: the AUG model
Source: Sci Rep. 2025 May 20;15:17552. doi: 10.1038/s41598-025-01150-8 (PMC12092762; doi:10.1038/s41598-025-01150-8)

A

Number of interactions

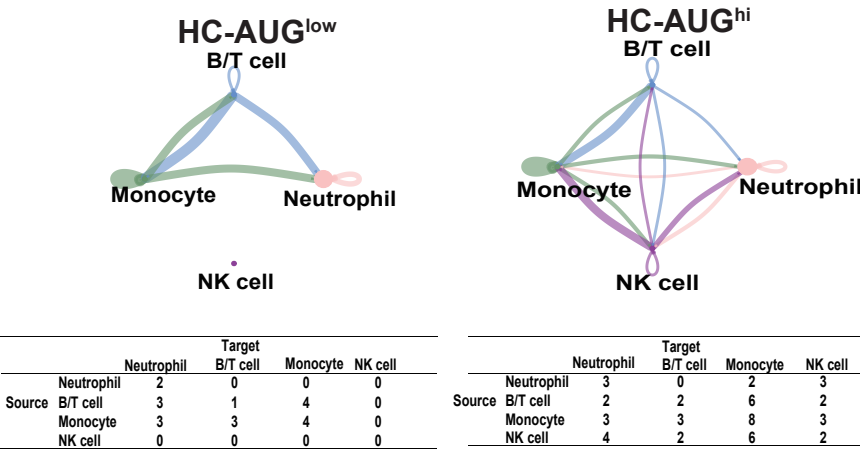

B

Interaction weights

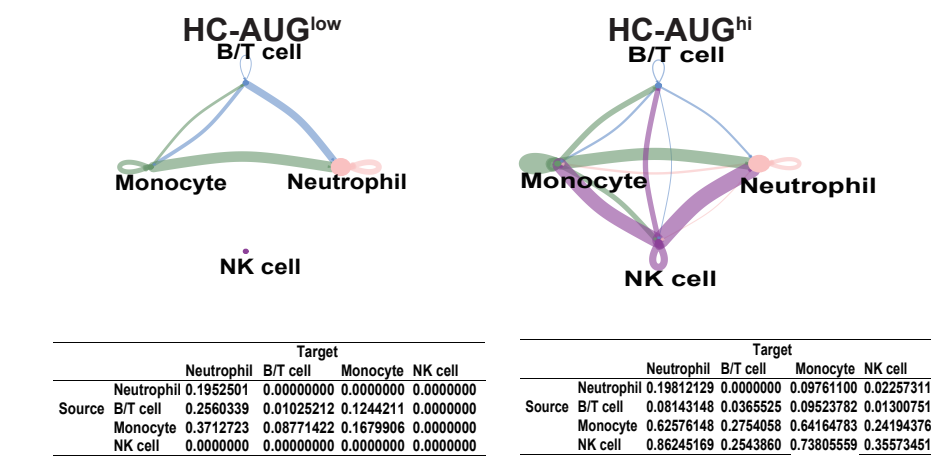

E

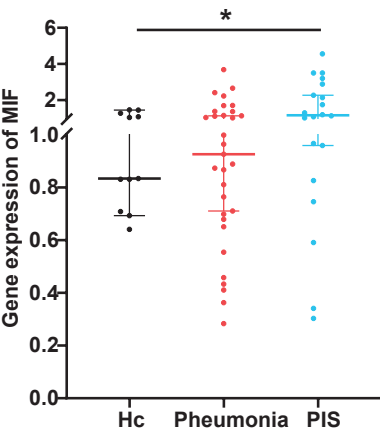

C

HC-AUG<sup>low</sup>

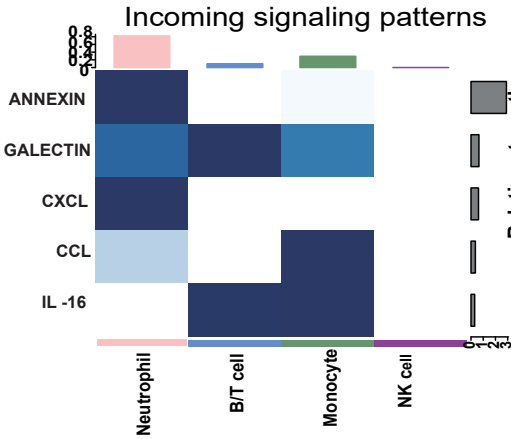

D

HC-AUG<sup>hi</sup>

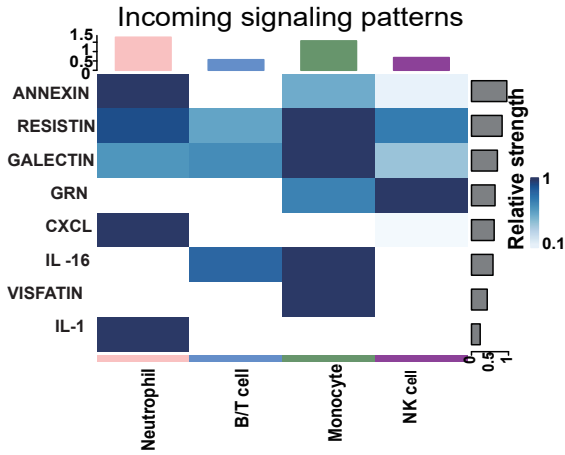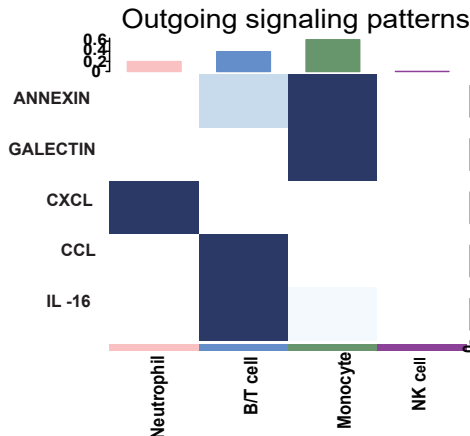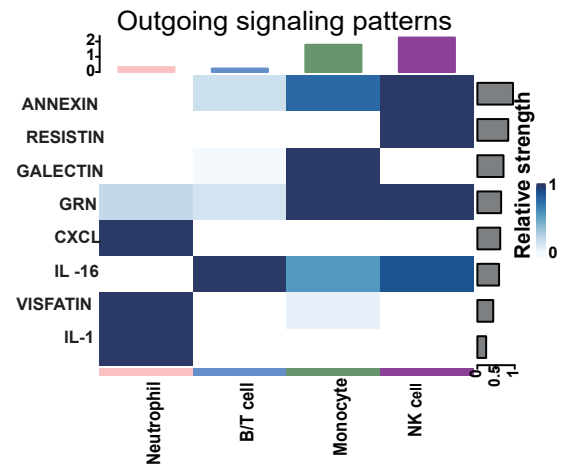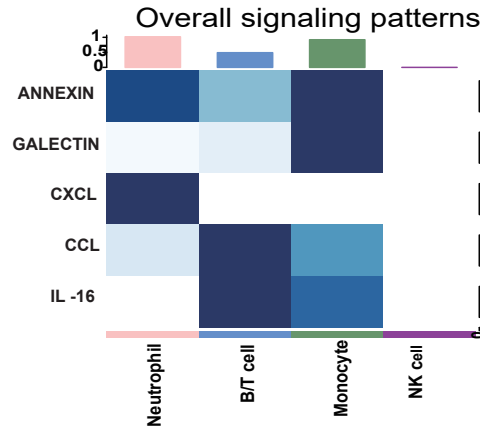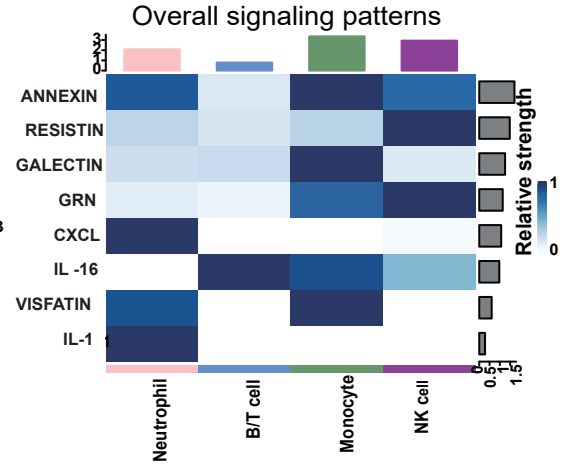

Supplement: Supplementary file 1 — Supplementary Information 1. [file 41598_2025_1150_MOESM1_ESM.pdf]
