# Supplementary material for: Sphingolipid metabolism-related genes as diagnostic markers in pneumonia-induced sepsis: the AUG model
Source: Sci Rep. 2025 May 20;15:17552. doi: 10.1038/s41598-025-01150-8 (PMC12092762; doi:10.1038/s41598-025-01150-8)
Supplement: Supplementary file 2 — Supplementary Information 2. [file 41598_2025_1150_MOESM2_ESM.docx]

**Supplementary figure legend**

**Figure S1. Analysis of enhanced immune cell communications in healthy controls with high AUG expression.**

**A-B.** Analysis of intercellular communication numbers (A) and weights (B) in healthy controls (HC) with low (HC-AUG^low^) and high (HC-AUG^hi^) AUG expression levels. Arrows denoted the direction of signal transmission from signaling to receiving cells. **C-D.** Comparative analysis of incoming, outgoing, and overall signaling patterns across different cell subsets in HC-AUG^low^ (C) and HC-AUG^hi^ groups (D). Upper square bar graphs indicated communication strength within specific pathways, while grey bar graphs represented the number of receptor-ligand pairs. **E.** Analysis of MIF gene expression in our clinical cohort revealed significantly elevated levels in PIS patients compared to healthy controls (HC). Data were analyzed using Bonferroni-adjusted Mann-Whitney tests for all intergroup comparisons (HC vs Pneumonia vs PIS), with significance denoted as *p<0.05, **p<0.01, ***p<0.001, ****p<0.0001.
