# Supplementary material for: Sphingolipid metabolism-related genes as diagnostic markers in pneumonia-induced sepsis: the AUG model
Source: Sci Rep. 2025 May 20;15:17552. doi: 10.1038/s41598-025-01150-8 (PMC12092762; doi:10.1038/s41598-025-01150-8)
Supplement: Supplementary file 3 — Supplementary Information 3. [file 41598_2025_1150_MOESM3_ESM.docx]

**Table S1. Sphingolipid metabolism-related genes from KEGG.**

| SYMBOL | ENTREZID | GENENAME |
| --- | --- | --- |
| SPTLC1 | 10558 | serine palmitoyltransferase long chain base subunit 1 |
| SPTLC2 | 9517 | serine palmitoyltransferase long chain base subunit 2 |
| SPTLC3 | 55304 | serine palmitoyltransferase long chain base subunit 3 |
| KDSR | 2531 | 3-ketodihydrosphingosine reductase |
| CERS1 | 10715 | ceramide synthase 1 |
| CERS2 | 29956 | ceramide synthase 2 |
| CERS4 | 79603 | ceramide synthase 4 |
| CERS3 | 204219 | ceramide synthase 3 |
| CERS6 | 253782 | ceramide synthase 6 |
| CERS5 | 91012 | ceramide synthase 5 |
| ASAH1 | 427 | N-acylsphingosine amidohydrolase 1 |
| ASAH2 | 56624 | N-acylsphingosine amidohydrolase 2 |
| ACER2 | 340485 | alkaline ceramidase 2 |
| ACER1 | 125981 | alkaline ceramidase 1 |
| ACER3 | 55331 | alkaline ceramidase 3 |
| DEGS1 | 8560 | delta 4-desaturase, sphingolipid 1 |
| DEGS2 | 123099 | delta 4-desaturase, sphingolipid 2 |
| SGMS1 | 259230 | sphingomyelin synthase 1 |
| SGMS2 | 166929 | sphingomyelin synthase 2 |
| SMPD1 | 6609 | sphingomyelin phosphodiesterase 1 |
| SMPD2 | 6610 | sphingomyelin phosphodiesterase 2 |
| SMPD3 | 55512 | sphingomyelin phosphodiesterase 3 |
| SMPD4 | 55627 | sphingomyelin phosphodiesterase 4 |
| ENPP7 | 339221 | ectonucleotide pyrophosphatase/phosphodiesterase 7 |
| CERK | 64781 | ceramide kinase |
| PLPP1 | 8611 | phospholipid phosphatase 1 |
| PLPP3 | 8613 | phospholipid phosphatase 3 |
| PLPP2 | 8612 | phospholipid phosphatase 2 |
| SGPP1 | 81537 | sphingosine-1-phosphate phosphatase 1 |
| SGPP2 | 130367 | sphingosine-1-phosphate phosphatase 2 |
| SPHK1 | 8877 | sphingosine kinase 1 |
| SPHK2 | 56848 | sphingosine kinase 2 |
| SGPL1 | 8879 | sphingosine-1-phosphate lyase 1 |
| UGCG | 7357 | UDP-glucose ceramide glucosyltransferase |
| GBA | 2629 | glucosylceramidase beta 1 |
| GBA2 | 57704 | glucosylceramidase beta 2 |
| B4GALT6 | 9331 | beta-1,4-galactosyltransferase 6 |
| B4GALT5 | 9334 | beta-1,4-galactosyltransferase 5 |
| GLB1 | 2720 | galactosidase beta 1 |
| UGT8 | 7368 | UDP glycosyltransferase 8 |
| GALC | 2581 | galactosylceramidase |
| GAL3ST1 | 9514 | galactose-3-O-sulfotransferase 1 |
| ARSA | 410 | arylsulfatase A |
| NEU1 | 4758 | neuraminidase 1 |
| NEU3 | 10825 | neuraminidase 3 |
| NEU4 | 129807 | neuraminidase 4 |
| NEU2 | 4759 | neuraminidase 2 |
| GLA | 2717 | galactosidase alpha |
| B4GALNT1 | 2583 | beta-1,4-N-acetyl-galactosaminyltransferase 1 |
| HEXA | 3073 | hexosaminidase subunit alpha |
| HEXB | 3074 | hexosaminidase subunit beta |
| PSAP | 5660 | prosaposin |
| PSAPL1 | 768239 | prosaposin like 1 |

**Table S2. Demographic Characteristics of Enrolled Cases.**

| **Characteristics** | **Group** | | | ***P*-value** |
| --- | --- | --- | --- | --- |
|  | **Healthy Control**  **(n = 11)** | **Pneumonia**  **(n = 31)** | **PIS**  **(n = 20)** |  |
| **Age, years old (median, IQR)** | 52.6 (35.8 - 69.4) | 61.4 (47.4 - 75.3) | 65.5 (53.7 - 77.2) | 0.06 |
| **≥ 60** | 4 (36.36) | 15 (48.39) | 15 (75.00) | 0.07 |
| **Gender** |  |  |  |  |
| **Male, n (%)** | 3 (27.27) | 17 (54.84) | 12 (60.00) | 0.19 |
| **Underlying conditions, n (%)** |  |  |  |  |
| **Cardiovascular diseases** | 1 (9.09) | 5 (16.13) | 7 (35.00) | 0.20 |
| **Cerebral infarction** | 0 (0.00) | 0 (0.00) | 2 (10.00) | 0.13 |
| **Diabetes** | 0 (0.00) | 1 (3.23) | 3 (15.00) | 0.29 |
| **Gastrointestinal diseases** | 1 (9.09) | 3 (9.68) | 5 (25.00) | 0.28 |
| **Lung cancer surgery** | 1 (9.09) | 1 (3.23) | 1 (5.00) | 0.75 |

**Table S3. Sequences of primers used in RT-qPCR experiments.**

| **Gene** | **Sequence (5’ – 3’)** | |
| --- | --- | --- |
|  | **Forward** | **Reverse** |
| **UGCG-Human** | ATGGCGCTGCTGGACCTGGCCTTG | TACATCTAGGATTTCCTCTGCTGTACCCCCACAGCGTAA |
| **GBA-Human** | ATGGAGTTTTCAAGTCCTTCCAGAGAGGAA | CTGGCGACGCCACAGGTAGGTGTGAAT |
| **ACER3-Human** | ATGGCTCCGGCCGCGGACC | ATGCTTCCTGAGAGGCTCAAACAGGATCACTGG |

**Table S4. The mRNA levels of AUG genes in clinical samples.**

| **Gene** | **Group** | | |
| --- | --- | --- | --- |
|  | **Healthy Control**  **(n = 11)** | **Pneumonia**  **(n = 31)** | **PIS**  **(n = 20)** |
| **ACER3 (Median, 95%CI)** | 0.87 (0.53 - 1.51) | 4.01 (3.44 - 5.29) | 7.39 (5.70 - 10.49) |
| **UGCG (Median, 95%CI)** | 0.85 (0.61 - 1.69) | 4.85 (3.18 - 7.08) | 10.91 (4.56 - 15.02) |
| **GBA (Median, 95%CI)** | 1.29 (0.19 - 2.39) | 5.07 (2.80 - 8.34) | 11.90 (4.21 - 33.41) |

**Table S5. The serum levels of AUG enzymes in clinical samples.**

| **Gene** | **Group** | | |
| --- | --- | --- | --- |
|  | **Healthy Control**  **(n = 11)** | **Pneumonia**  **(n =31)** | **PIS**  **(n = 20)** |
| **ACER3 (Median, 95%CI)** | 12.16 (11.17- 35.52) | 12.40 (12.07 -15.03) | 11.65 (11.27 - 19.33) |
| **UGCG (Median, 95%CI)** | 0.73 (0.13 - 3.00) | 3.44 (2.08 - 6.30) | 9.53 (3.32 - 11.74) |
| **GBA (Median, 95%CI)** | 0.31 (0.17 - 0.84) | 0.87 (0.73 - 2.56) | 1.84 (1.18 - 5.10) |
